# Ozoniferous Essence

**Published:** 1886-03

**Authors:** 


### OZONIFEROUS ESSENCES AS ANTICEPTICS.

Listerine possesses essential properties analogous in their effects to the ozoniferous ethers so highly recommended by Dr. Benjamin Ward Richardson and others as deodorizers and disinfectants for the sick room, and should be used in the same way—sprinkled over handkerchiefs, garments and the bed linen of fever cases. Mantegazza, "On the Action of Essences and Flowers in the Production of Atmospheric Ozone, and on their Hygienic Utility" (*Rendiconti del Reale Instituto Lombardo*, vol. iii., fasc. vi.), as quoted by Fox on Ozone, reports that the disciples of Empedocles were not in error when they planted aromatic and balsamic herbs as preventives of pestilence. He contends that a large quantity of ozone is discharged by odoriferous flowers, and that flowers destitute of perfume do not produce it. Cherry-laurel, clove, lavender, mint, lemon, fennel, etc., are plants which develop ozone largely on exposure to the sun's rays. Among flowers, the narcissus, heliotrope, hyacinth and mignonette are conspicuous; and of perfumes similarly exposed, eau-de-cologne, oil of bergamot, extract of millefleurs, essence of lavender and some aromatic tinctures. He also points out that the oxidation of the essential oils, such as nutmeg, aniseed, thyme, peppermint, etc., are convenient sources of ozone, and concludes that the ozoniferous properties of flowers reside in their essences, the most ozoniferous yielding the largest amount of ozone. It is of such aromatic essences that Listerine is composed, and hence its efficacy under the circumstances indicated.—*Sanitarian.*

